# *Abarema cochliacarpos* Extract Decreases the Inflammatory Process and Skeletal Muscle Injury Induced by *Bothrops leucurus* Venom

**DOI:** 10.1155/2014/820761

**Published:** 2014-07-20

**Authors:** Jeison Saturnino-Oliveira, Daiana Do Carmo Santos, Adriana Gibara Guimarães, Antônio Santos Dias, Marcelo Amorim Tomaz, Marcos Monteiro-Machado, Charles Santos Estevam, Waldecy De Lucca Júnior, Durvanei Augusto Maria, Paulo A. Melo, Adriano Antunes de Souza Araújo, Márcio Roberto Viana Santos, Jackson Roberto Guedes da Silva Almeida, Rita de Cássia Meneses Oliveira, Aldeidia Pereira de Oliveira, Lucindo José Quintans Júnior

**Affiliations:** ^1^Departamento de Fisiologia, Laboratorio de Farmacologia Pré-Clinica, Universidade Federal de Sergipe, SE, Brazil; ^2^Departamento de Morfologia, Laboratório de Biologia Celular e Estrutura, Universidade Federal de Sergipe, SE, Brazil; ^3^Programa de Pós-Graduação em Biotecnologia (RENORBIO), Universidade Federal de Sergipe, SE, Brazil; ^4^Departamento de Fisiologia, Laboratório de Bioquímica e Química de Produtos Naturais, SE, Brazil; ^5^Laboratório de Farmacologia das Toxinas, ICB, UFRJ, Universidade Federal do Rio de Janeiro, RJ, Brazil; ^6^Instituto Butantan, Laboratório de Ciências Fisiológicas e Química, São Paulo, SP, Brazil; ^7^Departamento de Farmácia, Laboratório de Ensaios e de Toxicidade Farmacêutica, Universidade Federal de Sergipe, SE, Brazil; ^8^Colegiado de Ciências Farmacêuticas, Universidade Federal do Vale do São Francisco, PE, Brazil; ^9^Departamento de Biofísica e Fisiologia, Universidade Federal do Piaui, Teresina, PI, Brazil; ^10^Laboratory Preclinical Pharmacology of Natural Products, Department of Physiology, Federal University of Sergipe, S/N Marechal Rondon Avenue, 49.100-000 São Cristovão, SE, Brazil

## Abstract

Snakebites are a public health problem, especially in tropical countries. However, treatment with antivenom has limited effectiveness against venoms' local effects. Here, we investigated the ability of *Abarema cochliacarpos* hydroethanolic extract (EAc) to protect mice against injection of *Bothrops leucurus* venom. Swiss mice received perimuscular venom injection and were subsequently treated orally with EAc in different doses. Treatment with EAc 100, 200, and 400 mg/kg reduced the edema induced by *B. leucurus* in 1%, 13%, and 39%, respectively. Although lower doses showed no antihypernociceptive effect in the Von Frey test, the higher dose significantly reduced hyperalgesia induced by the venom. Antimyotoxic activity of EAc was also observed by microscopy assessment, with treated muscles presenting preserved structures, decreased edema, and inflammatory infiltrate as compared to untreated ones. Finally, on the rotarod test, the treated mice showed better motor function, once muscle fibers were preserved and there were less edema and pain. Treated mice could stand four times more time on the rotating rod than untreated ones. Our results have shown that EAc presented relevant activities against injection of *B. leucurus* venom in mice, suggesting that it can be considered as an adjuvant in the treatment of envenomation.

## 1. Introduction

Accidents with venomous snakes represent a significant health problem, especially in tropical countries, where they frequently affect young and economically active men working in the countryside. In Brazil, most accidents are caused by snakes belonging to* Bothrops* genus, which induce extensive local damage, such as myonecrosis and edema [1, 2]. Particularly, the snake* Bothrops leucurus* is present in the Northeastern region of Brazil [3], being related to accidents with rural workers who often have difficult access to health services to receive the antiophidic treatment proposed by the Health Ministry, that is, the antibothropic antivenom.

Antibothropic antivenom is the only official treatment available, but it has low and limited effectiveness against the local effects of venoms [1]. The antivenom therapy is often applied late after the accident, when tissue destruction is already in process, potentially causing irreversible and disabling damage [4–6]. The use of plants and other alternative approaches to halt the effect of snake venoms or to accelerate tissue recovery has been proposed by previous studies [7–11]. Therefore, our group has been particularly concerned with the search for new and effective pharmacologically active principles from plants used in folk medicine to treat or prevent damage caused by accidents with venomous snakes.

Many traditional communities of northeastern Brazil make use of the bark of* Abarema cochliacarpos* in popular medicine.* A. cochliacarpos* is an ornamental tree native to Brazil, occurring mainly in the Atlantic Forest and in the Caatinga biomes. It belongs to the Mimosaceae family, being popularly known as “barbatimão” [12, 13]. An ethnopharmacological survey accomplished in a rural community in the Caatinga in the state of Sergipe, northeastern Brazil, identified popular applications of the bark of* A. cochliacarpos *[12]. In this community, the decoction of the bark is used to wash external ulcers while its tincture, made by placing the bark in the Brazilian beverage known as “cachaça,” is used against inflammation and gastric ulcers, among other uses [12, 13]. Other authors also observed similar applications in different traditional communities [14, 15]. According to previous studies, the hydroethanolic extract presents phenolics such as aurones, catechins, chalcones, flavanols, flavones, flavonols, leucoanthocyanidins, tannins and xanthones, besides saponins, and steroids [16].

In our study, we assessed the antiophidic ability of* A. cochliacarpos* extract, in order to propose a new option for the treatment of envenoming, besides the antivenom, by using a plant that is abundant in Brazil, and compared the extract activity with dexamethasone, a steroidal anti-inflammatory drug previously described to be active against some* Bothrops* venoms effects [11].

## 2. Material and Methods

### 2.1. Material

*B. leucurus* snake venom was obtained from CEPLAC (Comissão Executiva do Plano da Lavoura Cacaueira, Bahia, Brazil).* B. leucurus* venom and hydroethanolic extract of* A. cochliacarpos* were dissolved in physiological saline solution (PSS); PSS was composed of (mM) NaCl, 135; KCl, 5; CaCl_2_, 2; MgCl_2_, 1; NaHPO_4_, 1; NaHCO_3,_ 15, and dextrose, 11. The pH of this solution was equilibrated to 7.3 with 5% CO_2_/95% O_2_. Dexamethasone was obtained from Aché (São Paulo, Brazil).

### 2.2. Plant Material and Extract Preparation

*Abarema cochliacarpos* (Gomes) Barneby & Grimes stem barks were collected in São Cristóvão, state of Sergipe, Brazil (230 m, 11°01′63.2′′ south, 37°15′86.6′′ west). The plant material was identified by Dr. Ana Paula Prata and a voucher specimen was deposited at the herbarium of the Federal University of Sergipe under the number ASE 014639. Plant material (5 kg) was dried at 37°C with air circulation and renewal until complete dehydration. Then, it was reduced to powder and subsequently subjected to extraction in 90% ethanol for 5 days with exhaustive maceration. After this period, the extract was filtered and concentrated in a rotary evaporator under reduced pressure at 50°C yielding 533.4 g of hydroethanolic extract. The phytochemical analysis of* A. cochliacarpos* has been recently performed and described [16].

### 2.3. Animals

Male Swiss mice (25.0 ± 5.0 g), 2-3 months of age, were used throughout this study. The animals originated from the Central Animal Care of the Federal University of Sergipe. The animals were randomly housed in appropriate cages at 22 ± 2°C on a 12 h light/dark cycle with free access to food and water. Experimental protocols were approved by the Animal Care and Use Committee (CEPA/UFS number 10/11) at the Federal University of Sergipe.

### 2.4. Experimental Design

Mice were divided into six groups of 6 animals. They were anesthetized with ketamine (100 mg/kg) and xylazine (10 mg/kg) and then injected with crude venom of* Bothrops leucurus* (BlV) 1.0 mg/kg in PSS by applying 50 *μ*L of the solution next to the* extensor digitorum longus* (EDL) muscle of the right hind limb (EDL perimuscular injection, in order to prevent direct mechanical damage to the muscle), as described previously [9, 17]. Mice treated with* A. cochliacarpos* received administration by oral gavage.

*Group I *(control group). Mice were not subjected to muscle injury induced by venom and instead they received PSS in order to check for any changes in the parameters analyzed. 

*Group II *(BlV group). Mice received 1.0 mg/kg of* B. leucurus* venom injection (50 *μ*L) into the right paw. 

*Group III *(BlV + Dexamethasone – Dexa). Five minutes after venom injection, intravenous dexamethasone (2 mg/kg) was administered. 

*Group IV *(BlV + EAc 100 mg/kg). Five minutes after venom injection, mice received hydroethanolic extract of* A. cochliacarpos* by oral gavage (EAc, 100 mg/kg in 100 *μ*L). 

*Group V *(BlV + EAc 200 mg/kg). Five minutes after venom injection, mice received hydroethanolic extract of* A. cochliacarpos* by oral gavage (EAc, 200 mg/kg in 100 *μ*L). 

*Group VI *(BlV + EAc 400 mg/kg). Five minutes after venom injection, mice received hydroethanolic extract of* A. cochliacarpos* by oral gavage (EAc, 400 mg/kg in 100 *μ*L). 

### 2.5. Edematogenic Activity

The induction of edema was evaluated in all groups. Measurements were made at 0, 15, 30, 60, and 90 min after venom injection. An analog caliper rule was used to measure the mediolateral and anteroposterior widths of the paw, and the product of these values is reported as mm^2^.

### 2.6. Motor Functional Activity: Rotarod Test

Motor activity was assessed using the rotarod test to analyze the riding time as previously described [9]. The mice were trained daily for a period of 120 s for 5 days on the rotating cylinder (8 rpm) (Rota Rod EFF 412, Insight, Ribeirão Preto, SP, Brazil). One, three, and seven days after injection of 1.0 mg/kg venom alone or with treatments, the animals were submitted to the rotarod test and the time spent by the animal on the apparatus was recorded. Each animal underwent three trials, with an interval of at least 2 h for all animals, and the mean time spent on the rod was determined for each group.

### 2.7. Hyperalgesia Induced by* B. leucurus* Venom

Mechanical sensation of the hind paw as an index of mechanohyperalgesia test was assessed by pressure stimulation method (Von Frey modified method), as described by Mogil et al. [18]. Briefly, the nociceptive threshold was measured at different times after venom injection and treatments using an electronic anesthesiometer (EFF 301, Insight, Ribeirão Preto, SP, Brazil). A force (in g) of increasing magnitude was applied to the paw. When the animals reacted by withdrawing the paw, the force needed to induce such response was recorded and represented the nociceptive threshold.

### 2.8. Myotoxicity* In Vivo*

We evaluated the myotoxicity of* B. leucurus* venom by measuring the increase of plasma creatine kinase (CK) activity induced by intramuscular (i.m.) injection of venom alone or followed by i.v. dexamethasone or different doses of* A. cochliacarpos* extract by oral gavage. The venom was dissolved in PSS to a final volume of 0.1 mL (1.0 mg/kg) and they were injected into the rear thigh of the mice. Negative controls consisted of mice injected with the same volume of PSS.

### 2.9. Muscle Damage Histological Analysis

#### 2.9.1. Light Microscopy

Twenty-four hours after the venom injection and respective treatments, the animals were killed under anesthesia with ethyl ether; their EDL muscles were gently removed, fixed in standard paraformaldehyde, embedded in paraffin, longitudinally sectioned, and stained with hematoxylin and eosin (HE) for light microscopy analysis.

#### 2.9.2. Scanning Electron Microscope (SEM)

Some mice had their EDL muscles studied by scanning electron microscopy after the removal of connective tissue matrices using collagenase. Muscles were rinsed twice in physiological buffer solution (PBS) and fixed in 2.5% glutaraldehyde and 2% paraformaldehyde in phosphate buffer (0.1 M, pH 7.4) overnight at room temperature. After being washed three times in PBS, samples were fixed in 1% osmium tetroxide in aqueous solution (pH 7.4) at 4°C for 1 h. They were then dehydrated, dried in a critical point dryer (CPD 030, Balzers, Ribeirão Preto, SP, Brazil), and gold-sputtered (SCD 040, Balzers, Ribeirão Preto, SP, Brazil). Scanning electron microscope (SEM) images were taken on a LEO 435 VP SEM, 30 kV used to image capture (Carl Zeiss, Oberkochen, Germany).

### 2.10. Statistical Analysis

Data were expressed as mean ± SEM, and Student's* t*-test was used for statistical analysis. The *P* values <0.05 and <0.01 were used to indicate a significant difference between means.

## 3. Results

**Figure 1:**
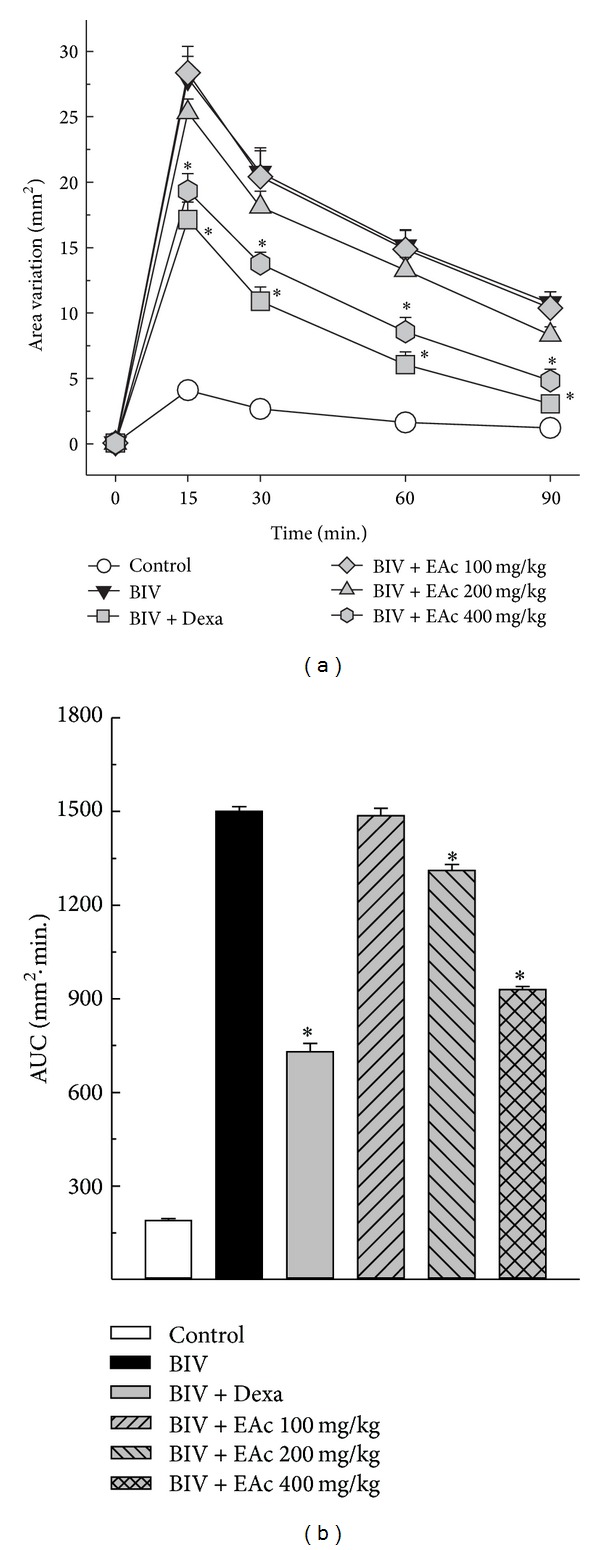
Effect of* A. cochliacarpos* extract (EAc) on* B. leucurus* venom's edematogenic activity. Mice received perimuscular injection of PSS or* B. leucurus *venom (1 mg/kg). The animals were treated with oral EAc at 100 mg/kg, 200 mg/kg, and 400 mg/kg and i.v. dexamethasone (2 mg/kg) 5 min after venom injection. Results show hind limb edema measured with a caliper rule until 90 minutes after venom injection (panel (a)). Panel (b) shows the area under the curve analysis with the data observed in (a). Data report means ± SEM (*n* = 6). *P < 0.05* versus* venom group (Student's *t*-test).

The venom of* B. leucurus* (1.0 mg/kg) showed edematogenic effect after perimuscular injection (Figure 1(a)). Fifteen minutes after injection, the venom induced a variation in leg area of 27.97 ± 2.44 mm^2^, while in the control group the variation caused by the injection of PSS was of only 4.10 ± 0.59 mm^2^. The limb edema induced by the venom was partially inhibited by increasing doses of* A. cochliacarpos* extract and by dexamethasone (Figures 1(a) and 1(b)). Treatment with dexamethasone (2.0 mg/kg) reduced the overall edema in 52%, while 100, 200, and 400 mg/kg* A. cochliacarpos* extract reduced the edema induced by* B. leucurus *in 1%, 13%, and 39%, respectively (Figure 1(b)).

**Figure 2:**
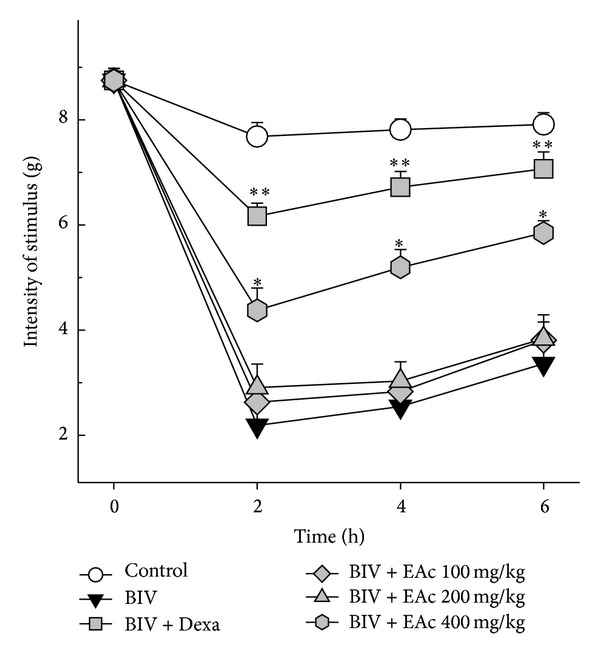
Effect of* A. cochliacarpos* extract (EAc) on* B. leucurus* venom's mechanical hyperalgesia response. Mice received perimuscular injection of PSS or* B. leucurus *venom (1 mg/kg). The animals were treated with oral EAc at 100 mg/kg, 200 mg/kg, and 400 mg/kg and i.v. dexamethasone (2 mg/kg) 5 min after venom injection. Results showing the force needed to cause paw withdraw (pain threshold). Data report means ± SEM (*n* = 6). *P < 0.05 and **P < 0.01* versus* venom group (Student's *t*-test).

The injection of BlV (1 mg/kg) into the mice hind limbs caused a significant decrease in nociceptive threshold (hyperalgesia), besides the increase in limb volume. In our protocol, the peak of the hypernociceptive response occurred 2 h after venom injection, and after this time the phenomenon started to decrease and completely disappeared at 24 h (data not shown). When animals were treated with 400 mg/kg* A. cochliacarpos* extract or with dexamethasone, the hyperalgesic response was significantly reduced. Lower doses of the extract showed no antihyperalgesia effect (Figure 2).

**Figure 3:**
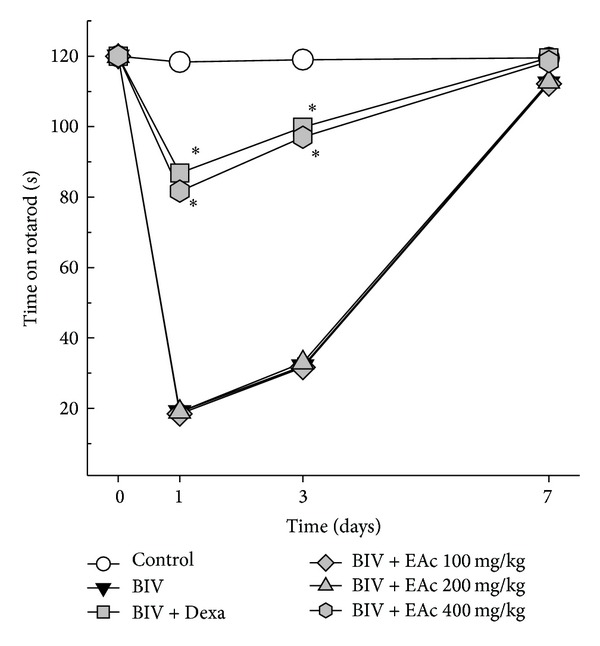
Functional activity. Time spent by mice on the rotarod (8 rpm) before and after receiving* B. leucurus* venom (1.0 mg/kg). The animals were treated with oral EAc at 100 mg/kg, 200 mg/kg, and 400 mg/kg and i.v. dexamethasone (2 mg/kg) 5 min after venom injection. Time zero represents data obtained before venom injection. Data report means ± SEM (*n* = 6). *P < 0.05* versus* venom group (Student's *t*-test).

After* B. leucurus* venom injection, all animals, including those receiving treatment, showed a decrease in functional ability to stand on the rotarod. However, on the first and third days after injection, mice receiving venom only or venom treated with 100 mg/kg and 200 mg/kg EAc showed a more pronounced decrease, compared to animals treated with Dexa or 400 mg/kg EAc. This result shows that EAc decreased the impact of venom on motor functional activity. By day 7, all animals, including those that received only venom injection without any treatment, were able to stand on the rotarod as long as the control mice, showing recovered muscle function (Figure 3).

**Figure 4:**
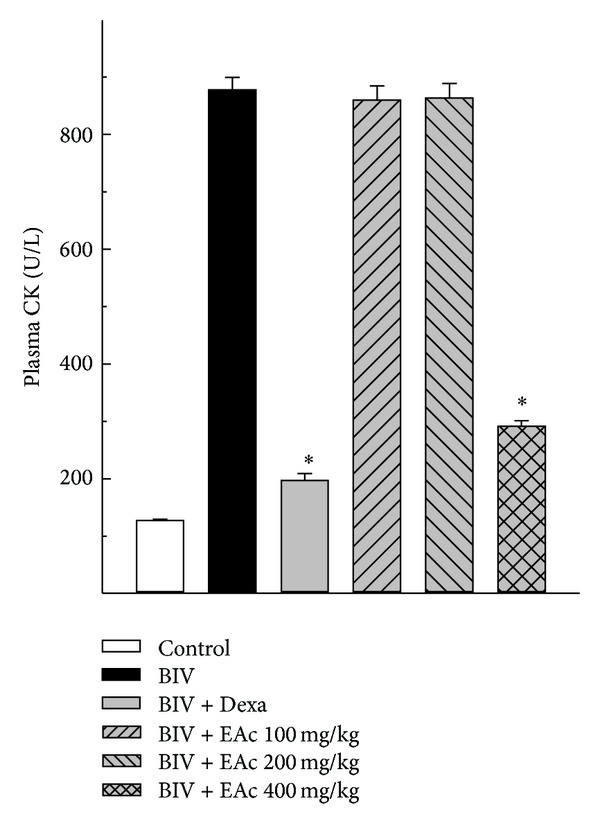
*In vivo* myotoxicity. The figure shows the effect of EAc and dexamethasone on* B. leucurus *myotoxic activity* in vivo*. Results show plasma CK activity 2 hours after injection of the venom alone or followed by treatment with i.v. dexamethasone or EAc by oral gavage. Data report means ± SEM (*N* = 5). *P < 0.05 for the difference between treated groups and the venom (Student's *t*-test).

Mice injected intramuscularly with the venom of* B. leucurus* (1 mg/kg) presented, 2 hours after venom injection, an increased activity of CK in plasma, which ranged from 127.75 ± 1.59 U/L (*n* = 5) in the group receiving the PSS solution up to 877.70 ± 21.54 U/L (*n* = 5) in the group receiving the venom. Oral treatment of mice with the different doses of EAc partially inhibited the* in vivo* myotoxic activity of the venom (Figure 4).

**Figure 5:**
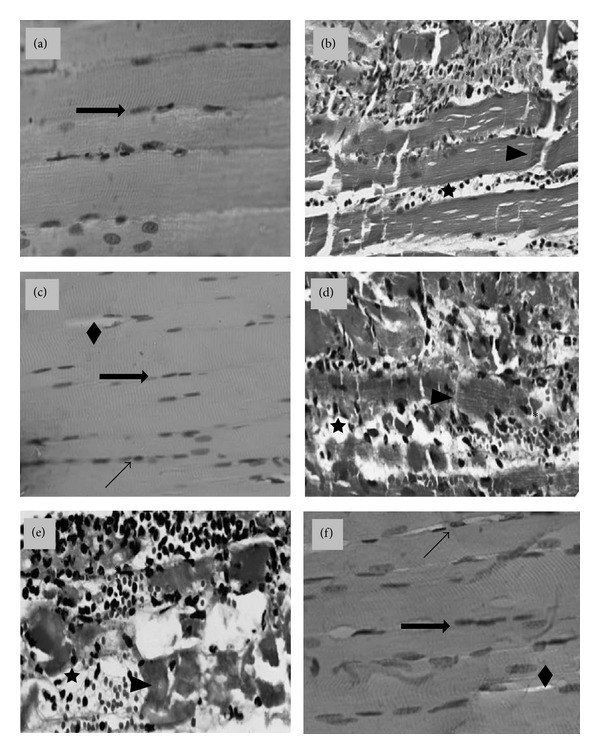
Light microscopy of mouse EDL muscle 24 h after perimuscular injection of* B. leucurus *venom: effect of* A. cochliacarpos* extract (EAc). Longitudinal sections stained with hematoxylin and eosin: (a) PSS; (b) venom (1.0 mg/kg); (c) venom + dexamethasone (2 mg/kg, i.v); (d) venom + EAc 100 mg/kg; (e) venom + EAc 200 mg/kg; and (f) venom + EAc 400 mg/kg (*n* = 6 per group). In panel (a), panoramic view of control muscle showing normal multinucleated myofibers with peripheral nuclei (arrow) and no morphological changes. Panels (b), (d), and (e) show necrotic myofibers in different stages of degeneration (arrowheads), edema between the fibers (stars) and intense inflammatory infiltrate (asterisks) that reaches the internal portions of the muscle. In (c) and (f), observe preserved muscle fibers with peripheral nuclei (arrows). We can see some inflammatory cells (diagonal arrows) and small vacuoles (lozenges). Magnification: 40x.

Light microscopy of the EDL muscles 24 h after injection of* B. leucurus* venom showed structural disorganization of muscle fibers with cellular damage and inflammatory cellular infiltration, characteristics of a typical inflammatory reaction. Treatment with 400 mg/kg* A. cochliacarpos* extract and with dexamethasone preserved the muscle fibers and seemed to reduce the presence of inflammatory cells (Figure 5).

**Figure 6:**
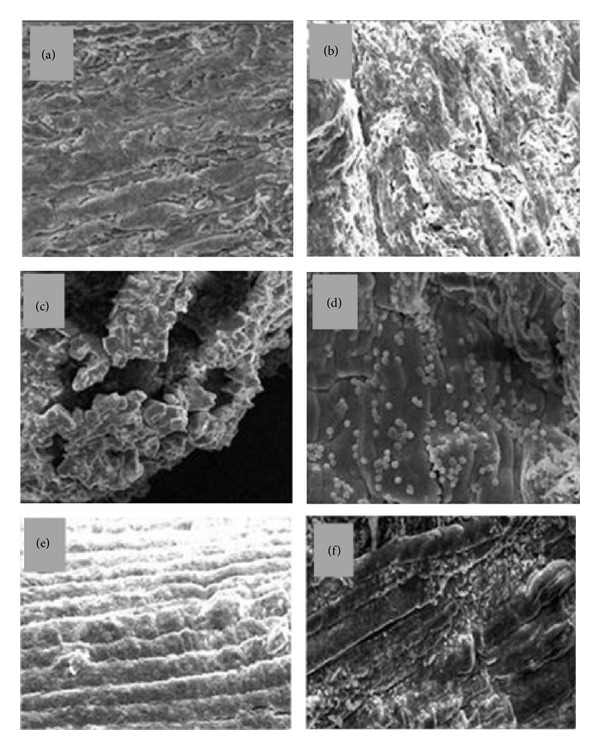
The cytoarchitecture of mouse EDL muscle 24 h after perimuscular injection of* B. leucurus *venom: effect of* A. cochliacarpos* extract (EAc). Muscles were studied by scanning electron microscopy after the removal of connective tissue matrices using a modified KOH-collagenase digestion method. (a) PSS; (b), (c), and (d) venom (1.0 mg/kg); (e) venom + dexamethasone (2 mg/kg, i.v); (f) venom + EAc 400 mg/kg (*n* = 6 per group). Magnification: 100x ((a), (b), (e), and (f)); 400x ((c) and (d)).

Ultrastructural study of EDL muscle fibers confirmed the observations of light microscopy (Figure 6). Control muscles injected with PSS had a constant diameter of about 20 *μ*m and showed long cylindrical forms, occasionally splitting or dividing to provide branches within the muscle (Figure 6(a)). On the other hand, in the muscles injected with* B. leucurus* venom (1.0 mg/kg), focal areas of myonecrosis were abundant after 24 h. Injured fibers presented dilated perimysium and disoriented and condensed myofibrils (Figures 6(b) and 6(c)). Furthermore, as shown in Figure 6(d), hemorrhage was apparent in the endomysial connective tissue, and hemolysis was discernible. Degeneration was pronounced in areas where the erythrocytes were tightly packed between the muscle fibers. Myofibrils were hypercontracted leaving, as a consequence, areas of overstretched myofibrils as well as empty spaces. On its turn, muscles of mice treated with i.v. dexamethasone showed no damage or extensive hemorrhage (Figure 6(e)). Finally, the oral administration of* A. cochliacarpos* hydroalcoholic extract (400 mg/kg) also decreased the myonecrotic effect of* B. leucurus* venom, with fewer areas of hypercontracted myofilaments or hemorrhagic components (Figure 6(f)).

## 4. Discussion

Our study confirmed the ability of* Bothrops leucurus* venom injection to induce muscle damage, edematogenic activity, hyperalgesia, and motor function impairment. Among the important components of this venom are several metalloproteinases and phospholipases A_2_ (PLA_2_), which present cytotoxic and proinflammatory properties [3, 19–23]. Our results provide for the first time an experimental and scientific support for the use of* A. cochliacarpos* extractin cases of accidents with* B. leucurus *snake venom,which had some important activities inhibited in experimental conditions by the plant's crude extract.

Edema is an important effect of* Bothrops* venoms, which along with tissue necrosis can be severe enough to cause functional loss or even compartment syndrome [24]. As part of the classical inflammatory reaction, the edema generation was rapidly set following venom injection. Venom-induced edema has been described as an inflammatory process induced by components that involve local mediators derived from arachidonic acid and autacoids such as histamine and serotonin and is not substantially antagonized by antivenom [25, 26], constituting a major complication related to accidents caused by* Bothrops* in large animals. In agreement with previous studies [11, 27], the anti-inflammatory properties of dexamethasone were able to partially inhibit the edema induced by venom injection, strongly supporting that at least in part the venom activity depends on inflammation. Interestingly, the crude extract of* A. cochliacarpos* was also able to reduce the edematogenic effect of* B. leucurus* venom injection, confirming early observations showing that this plant presents some anti-inflammatory properties [16, 28–31].

Inflammation is frequently associated with pain and hypernociception [32, 33]. Inflammatory hyperalgesia follows alterations in transduction sensitivity in the peripheral terminals (nociceptors) and in excitability in the central nervous system. Such alterations are secondary to the activation of chemosensitive nociceptors by inflammatory mediators, which are extensively induced by snake venom injection [33–35]. It has been shown that hyperalgesia induced by* Bothrops* sp. venoms is not neutralized when antivenoms are administered after envenomation [35], and the clinical relevance of pain following envenomation points to the need to develop pain-controlling therapeutic strategies. The known antihyperalgesic actions of dexamethasone [36] were confirmed in our study. Again, oral administration of* A. cochliacarpos* extract successfully decreased the hyperalgesic action of* B. leucurus *venom.

As previously shown, i.m. injection of the* Bothrops* venoms induces extensive myonecrosis in mice [37–42]. Our study showed the important* in vivo* myotoxicity of* B. leucurus* venom, which was decreased by both dexamethasone and* A. cochliacarpos* extract, as well as the plasma CK activity induced by the venom. This is of great importance considering the high myotoxicity of* Bothrops* venoms and the lack of protection from this activity by the available treatment, the antibothropic antivenom, leading to functional disabilities due to poor muscle regeneration [43].

Local and systemic skeletal muscle degeneration is a common consequence of envenomations due to snakebites. PLA_2_s are important myotoxic components in* Bothrops* venoms, inducing a similar pattern of degenerative events in muscle cells. Myotoxic PLA_2_s bind to acceptors in the plasma membrane, which might be lipids or proteins and which may differ in their affinity for the PLA_2_s. Upon binding, myotoxic PLA_2_s disrupt the integrity of the plasma membrane by catalytically dependent or independent mechanisms, provoking a pronounced Ca^2+^ influx which, in turn, initiates a complex series of degenerative events associated with hypercontraction, activation of calpains and cytosolic Ca^2+^-dependent PLA_2_s, and mitochondrial Ca^2+^ overload [6, 44]. Our experiments showed dose-dependent inhibition of* B. leucurus* myotoxic activity by* A. cochliacarpos* extract, which could be related to inhibition of the venom's PLA_2_s, besides decreasing muscle damage by the inflammatory reaction itself.

Finally, mice receiving treatment with dexamethasone or* A. cochliacarpos* extract showed better functional performance, that is, because the morphological and biochemical properties seemed to be preserved, and once edema and hypernociception were to some extent prevented, their physical activity was superior to that achieved by untreated animals. Although in morphological analysis we did not measure the extent of the damaged area or the number of centrally located nuclei, this qualitative demonstration of the positive effects of* A. cochliacarpos* extract was confirmed by rotarod performance and the decreased plasma CK activity.

Several groups have demonstrated potential therapeutic uses of* A. cochliacarpos*, such as protective effects in acute experimental colitis [28], gastroprotective effects and wound-healing properties [29], and antioxidant and anti-inflammatory properties [16, 31]. Those abilities have been related to inhibition of oxidative stress and inflammatory responses via direct downregulation of nitric oxide (NO) and reactive oxygen species (ROS) generation, which could damage different cellular components such as lipids, proteins, and DNA. The antioxidant and anti-inflammatory properties of* A. cochliacarpos* hydroethanolic extract may be mediated, at least in part, by the presence of (+)-catechin, a flavonoid-type compound, the most abundant in the extract, which induce downregulation of proinflammatory cyclooxygenase-2 (COX-2) and inducible nitric oxide synthase (iNOS) enzymes [16, 28, 29, 31].

## 5. Conclusion

Our results have shown the antiophidic ability of* A. cochliacarpos* extract, which seem to be related to its antioxidant, antihyperalgesic, and anti-inflammatory properties, for the first time giving scientific support for its use in folk medicine as an adjuvant to the available antivenom therapeutics.
